# Characteristics of drug-resistant staphylococci isolated from milk of lambed ewes during the perinatal period

**DOI:** 10.2478/jvetres-2025-0014

**Published:** 2025-03-25

**Authors:** Agata Hahaj-Siembida, Aneta Nowakiewicz, Monika Greguła-Kania, Mariola Bochniarz, Aleksandra Trościańczyk, Marcelina Osińska

**Affiliations:** Department of^1^Sub-Department of Veterinary Microbiology, Department of Preclinical Veterinary Sciences, University of Life Sciences, 20-950 Lublin, Poland; Department of Animal Breeding and Agricultural Advisory, Faculty of Animal Sciences and Bioeconomy, University of Life Sciences, 20-950 Lublin, Poland

**Keywords:** *Staphylococcus*, sheep mothers, drug resistance, animal health

## Abstract

**Introduction:**

Staphylococci are still a challenge in veterinary medicine, as they are one of the aetiological factors causing clinical and subclinical mastitis in small ruminants. The aim of the study was to analyse the occurrence of staphylococci in milk obtained from Świniarka (SW) and Uhruska (UHR) sheep and to characterise their drug resistance and virulence.

**Material and Methods:**

In total, 50 milk samples were collected from ewes seven days after parturition. Drug susceptibility analysis was performed based on Clinical and Laboratory Standards Institute standards and demonstration of the presence of resistance genes was attempted.

**Results:**

*Staphylococcus* spp. strains were identified in 70% of the samples, and 57.2% of the strains were *S. aureus*. Most of the tested strains (88.6%) were resistant to at least one antimicrobial, and resistance to tetracycline and erythromycin was the dominant type in *S. aureus* strains. Coagulase-negative species (CoNS) exhibited resistance predominantly to penicillin, cefoxitin and tetracycline (86.6%, 73.3% and 46.6%, respectively).The presence of the *mec*C gene was detected in four cefoxitin resistant strains. In tetracycline- and chloramphenicol-resistant strains, phenotypic and genotypic resistance was statistically significantly more common among strains isolated from UHR than SW.

**Conclusion:**

The present study highlights the problem of potential subclinical mastitis caused by drug-resistant strains of *S. aureus* and other CoNS species in lambed ewes. However, the occurrence of virulence factors in these strains is very rare.

## Introduction

Bacteria of the *Staphylococcus* genus belong to a group of microorganisms occurring both as commensals of the mucous membranes of various species of animals and humans and as causative agents of many local and generalised infections of varying severity, ranging from mild or subclinical changes to life-threatening infections. This genus currently includes over 70 different species (27). Because of their relatively high resistance to unfavourable environmental conditions, such as low water activity, high temperatures, high sodium chloride concentration and low pH, staphylococci are able to survive outside the host for a relatively long time, constituting one of the most common pathogens contaminating food of animal origin. Numerous species belonging to the *Staphylococcus* genus are also components of the natural biota of the skin and mucous membranes of healthy sheep (30, 31). However, bacteria of the *Staphylococcus* genus are also the most common cause of infections in these animals, including those which develop into clinical and subclinical mastitis. The coagulase-positive species (CoPS) *S. aureus* is a particularly frequent aetiological agent of mastitis and is responsible for over 70% of cases, while a similar percentage of coagulase-negative species (CoNS) are isolated from cases of subclinical mastitis (29). The change in the status of a microorganism from a commensal to the causative agent of infection depends on many factors, including the way animals are kept and fed, the level of hygiene and biosecurity, milking practices and even climate changes (30). The breed of animal is also of great importance given the susceptibility to infections, anatomical condition of teats, number and size of offspring, length of the lactation period and typical prevalence of coexisting health problems which all vary from breed to breed (24). It has been shown that ewes of different breeds have different genetic predispositions to the development of clinical or subclinical mastitis (30).

Although sheep breeding has a long tradition in Poland, currently sheep constitute only 1% of the number of animals kept in our country and used for milk and meat. There are over a dozen breeds of sheep in specific or mixed types of use, with 17 native breeds covered by the Genetic Resources Protection Programme (19). These breeds include the Uhruska and Świniarka sheep examined in the current work.

The Uhruska Sheep (UHR) is a refined breed. This is a native variety developed in the region of centraleastern Poland as a cross between Merino ewes from the Poznań region and Leine and Kent rams. The animals make good use of farm feed and natural pastures. Sheep of this breed are medium in size and their body weight ranges from 55 to 60 kg. The Uhruska sheep is a meat breed with high fertility (140–160%) and high-quality wool (14).

The Świniarka Sheep (SW) is a primitive breed that is relatively resistant to diseases and unfavourable living conditions; the breed is undemanding in feeding (low-quality feed). These animals adapt perfectly to local environmental conditions. The representatives of this breed are small with a slight build and a body weight of 25–35 kg. The usability of this breed is low, and its milk production is only sufficient to feed the offspring. The reproduction of these sheep is characterised by seasonality and a high frequency of single litters. Sexual maturation is moderately late, at the age of 9–12 months, and fertility is estimated at 120% (14).

The analysis of factors that may potentially negatively affect the health status of these animals seems extremely important from the point of view of conservation and protection of the species. Both sheep breeds are considered endangered; moreover, the Świniarka breed currently requires conservation efforts (19).

The common occurrence of microorganisms of the *Staphylococcus* genus makes their contribution to the infection threat to sheep a serious one. Staphylococcal infections are associated with huge economic losses because of decreased milk production and poor milk quality. In addition, they negatively affect the welfare of both sheep and lambs and worsen lamb mortality. Infections may also cause fear, anxiety, changes in feeding behaviour and pain in affected ewes, which have adverse impacts on meat production (24).

Public health is an important issue regarding the consumption of products manufactured from raw materials originating from various breeds of sheep. The presence of staphylococci, especially of *S. aureus*, in consumed milk or milk products poses a significant threat to public health. This is related, on the one hand, to the increasing number of multidrug-resistant (MDR) isolates belonging to this group of microorganisms and, on the other hand, to the numerous virulence factors of *S. aureus* causing many life-threatening infections, such as those where its toxigenic mechanism is activated and leads to toxic shock syndrome (28). Increasing resistance usually results from the selection pressure associated with the frequent use of antimicrobials in the equally frequent infections in animals. There is usually a positive correlation between modified breeds with high milk or meat production and increased susceptibility to infections (31). When focusing on the presence of *Staphylococcus* in sheep, the “One Health” concept should not be ignored. A crucial aspect of this concept is food safety, including microbiological safety, which is determined by the health of food animals and necessarily by the health of sheep (22). Therefore, the aim of the study was to analyse the occurrence of *Staphylococcus* in the milk of lactating mothers of sheep from two different breeds and to characterise the strains in terms of their drug resistance and potential virulence.

## Material and Methods

The samples of milk were taken from two local native Polish breeds of sheep: SW and UHR. The animals were kept in the Bezek experimental station belonging to the University of Life Sciences in Lublin (51°11'60.00" N 23°15'60.00" E), located in south-eastern Poland. The basic herd numbers over 500 animals, including approximately 200 UHR dams and 50 SW dams. The ewes were fed according to the French Institut National de la Recherche Agronomique feeding system (according to their physiological status). All animals housed in the sheepfold were fed in the same way using the feed available on the farm. The animals were kept in the same building in uniform environmental conditions; however, the breeds were separated inside the building and during pasturing. The animals were kept in a closed system from September to mid-May and an open system with grazing from mid-May to early September. Mating lasted six weeks, starting in September for the UHR ewes and in October for the SW, and lambing lasted from mid-January to February for the UHR sheep and from February to March for the SW. The animals were of the same age, *i.e*. two years old.

### Milk sample collection, isolation and species identification

Milk samples were collected during a standard clinical examination conducted by a veterinarian between March and April 2023. Twenty-five animals with no clinical symptoms detected during the basic clinical examination of the udder (observation, palpation and comparison of both glands) were selected from each breed to provide the material. A total of 50 milk samples were collected as 25 samples from UHR and 25 from SW. Milk samples of 5 mL volume were collected aseptically from an udder half from each animal, on average seven days after parturition, according to a procedure described previously (25). In refrigeration conditions, the samples were transported to the laboratory within two hours, where 1 mL of each sample was cultured in nutrient broth medium supplemented with 6.5% NaCl (Biomaxima, Lublin, Poland) and incubated for 24 h at 37°C. Mannitol Salt LAB Agar and Baird-Parker LB (lysogeny broth) Agar (Biomaxima) were selected from the products for specific application to *Staphylococcus* spp. and used as selective media. Samples of 100 μL volume were streaked onto plates and incubated at 37°C for 24 h. One typical colony obtained from the media was selected for further analyses. Next, Gram staining (Gram Staining Kit, Merck KGaA, Darmstadt, Germany), catalase (3% hydrogen peroxide solution), and coagulase assays were performed (BBL Coagulase Plasma, Rabbit, with EDTA, BD, Franklin Lakes, NJ, USA). All catalase- and Gram-positive cocci were analysed using the matrix-assisted laser desorption/ionisation–time-of-flight mass spectrometry (MALDI TOF-MS) and coagulase-positive strains were further confirmed using the molecular method based on the analysis of *nuc* gene polymorphism as described previously (15). DNA was isolated from 24-h pure bacterial colonies using a commercial kit (Gram Plus and Yeast Genomic DNA Purification Kit; EURx, Gdańsk, Poland) in accordance with the manufacturer’s instructions. Amplification was performed according to the conditions described previously (14) and with Silver Taq Mix (Syngen Biotech, Teipei, Taiwan) and appropriately selected primers (Genomed, Warsaw, Poland) as described previously (15).

### Analysis of phenotypic antimicrobial susceptibility

The phenotypic antimicrobial susceptibility of *Staphylococcus* spp. was assessed using the disc-diffusion method on Mueller Hinton LAB-AGAR (Biomaxima Lublin, Poland). Thirteen discs with antibiotics (Oxoid, Basingstoke, UK) were used for this study: erythromycin 15 mg, cefoxitin 30 mg, gentamicin 10 mg, rifampicin 5 mg, quinupristin/dalfopristin 15 mg, clindamycin 2 mg (alone and in tests for the detection of inducible clindamycin-resistant *Staphylococcus* spp.), penicillin 10 IU, sulfamethoxazole/trimethoprim 25 mg, tetracycline 30 mg, nitrofurantoin 300 mg, linezolid 30 mg, ciprofloxacin 5 mg and chloramphenicol 30 mg. The procedure and interpretation of the results were performed in accordance with the Clinical and Laboratory Standards Institute (CLSI) guidelines (8, 9). The American Type Culture Collection *Staphylococcus aureus* ATCC 25923 reference strain was used for quality control, and the multidrug resistance of the strains was classified according to criteria described in a previous study (15).

### Detection of resistance and virulence genes

The *Staphylococcus* spp. strains isolated from the milk were tested for the presence of genes encoding phenotypic resistance. The following genes were selected: *mecA, mecC, blaZ* (resistance to β -lactams), *ermA, ermB, ermC, msrA* (resistance to macrolides), *cat pC223, cat pC221, cat pC194* (resistance to phenicols), *tetK, tetM, tetL* (tetracycline resistance), *acc(6′)-le* and *aph(3)-IIIa* (resistance to aminoglycosides). The sequences of the selected primers and the reaction conditions are described in previously published articles (10, 14). The *S. aureus* strains were also tested for the presence of the following virulence genes: *seA-E* (encoding enterotoxins), *tst* (encoding toxic shock syndrome toxin-1), *etA, etB* (encoding the ETA and ETB exfoliative toxins), *LukE-LukD* (responsible for leukotoxin *LUKE/LUKD*) and *PVL* (encoding leukocidin) (17).

DNA isolated according to the method described above (1 μL/20 μL of total volume of amplification reaction) and PCR reagent (PCR Mix Plus Red, A&A Biotechnology, Gdańsk, Poland) were used in all the analyses.

### Statistical analysis

The statistical analysis was performed using the Statistica 13.1 program (Dell Technologies,Round Rock, TX, USA). The Mann–Whitney U test (P-value < 0.05) was used to determine statistically significant differences between *S. aureus* and CoNS in terms of the level of phenotypic resistance and the occurrence of resistance genes and to compare the phenotypic resistance and the occurrence of resistance genes between strains isolated from the two breeds of sheep (P-value < 0.05).

## Results

### Isolation and identification of *Staphylococcus* species

MALDI TOF-MS confirmed that 35 isolates obtained from tested sheep milk samples belonged to the *Staphylococcus* genus. Among them, 20 strains (57%) were coagulase-positive and were all *S. aureus*, which was further confirmed by the results of the amplification of the *nuc* gene, and 15 strains (30%) were four different coagulase-negative species (*S. xylosus, S. simulans, S. condimenti* and *S. lentus*) (Table 1). A comparable positive rate of *Staphylococcus* isolation from the samples was observed in both animal breeds, with a slight predominance in the SW breed (54% *vs*. 46% of samples were positive, respectively).

**Table 1.** Occurrence of *Staphylococcus* species in milk by sheep breed

		*Staphylococcus aureus*	*S. xylosus*	*S. simulans*	*S. lentus*	*S. condimenti*
Breed	Uhruska (n = 16)	10	1	4		1
	Świniarka (n = 19)	10	7	1	1	
Total (n = 35)		20	8	5	1	1
%*		57.2	22.8	14.3	2.85	2.85

* – percentage of strains in relation to the total number of isolates

### Phenotypic drug susceptibility profiles of *Staphylococcus* species

Resistance to at least one antimicrobial was observed among most strains. Only four (11.4%) strains of the *S. aureus* species, originating from SW, were susceptible to all the tested antimicrobials. In turn, all the strains were susceptible to linezolid, nitrofurantoin and trimethoprim/sulfamethoxazole (Table 2). Additionally, no resistance of *S. aureus* to rifampicin was observed, and none of the CoNS strains was resistant to chloramphenicol, ciprofloxacin or streptogramins (Table 3). The highest percentage of resistance among *S. aureus* strains was recorded for tetracycline and erythromycin (35% each) and the next highest to chloramphenicol, penicillin and clindamycin (30% each). Constitutive resistance was noted for all the clindamycin-resistant strains. In terms of the breed of sheep providing a sample from which a resistant strain was isolated, in most cases, resistant *S. aureus* isolates originated from UHR, which also correlated with a higher proportion of MDR *S. aureus* strains from this breed, five coming from UHR and only one from SW. The profile of the CoNS strains was dominated by resistance to penicillin, cefoxitin and tetracycline (86.6%, 73.3%, and 46.6% of such strains, respectively) (Table 3), classifying of 1/3 (n = 5) of the CoNS strains as MDR (Table 2).

**Table 2.** Characteristics of *Staphylococcus* spp. strains isolated from Polish ewes of the Uhruska (n = 16) and Świniarka (n = 19) breeds

Breed	Species	Number of isolates	MDR	Phenotypic resistance profiles	Resistance and virulence gene profiles
Uhruska	*S. aureus*	1		R: P10	
		1		R: DA2	
		1		I: TE30, C30	*cat* (pC221), *tet*L
		1	5	R: P10, TE30, C30	*cat* (pC194)
				I: E15	
		1		I: E15	*mrs* A
		1	3	R: P10	
				I: QD15, DA2	
		1	3	I: TE30, CIP5, C30	*cat* (pC221), *tet*L, *tet*K
		1	4	I: E15, CN10, TE30, C30	*cat* (pC221), *cat* (pC194), *tet*L, *aph(3)-IIIa*, seB
		1		R: E15	*tet*L, *erm* A, *erm* B
				I: TE30, DA2,	
		1	4	R: FOX30, P10	*cat* (pC221), *cat* (pC194), *tet*L, *mec*C, *aph(3)-IIIa, αcc(6’)-Ie-*
				I: CN10, TE30, C30	
	*S. xylosus*	1		R: FOX 30, P10	
	*S. condimenti*	1		R: P10	
	*S. simulans*	1		R: TE30	*tet*K, *tet*L, *aph(3)-IIIa*
		1	3	R: FOX 30, P10, TE30	*tet*K
				I: DA2	
		1	4	R: FOX 30, P10, E15, TE30	*mec*C, *erm*B, *cat* (pC194), *tet*K, *tet*L, *aph(3)-IIIa, bla*Z
				I: DA2	
		1		R: TE30	*cat* (pC194)
Świniarka	*S. aureus*	4		susceptible	
		2		I: DA2	
		1		R: P10	
		1		R: P10	
				I: E15	
		1		I: E15	
		1	4	I: E15, DA2, TE30, C30	*cat* (pC221), *tet*L
		3		R: FOX30, P10	
	*S. xylosus*	1		R: P10	*tet*M, *aph(3)-IIIa, bla*Z

		1		R: FOX30, P10	*tet*M, *mec*C, *aph(3)-IIIa, αcc(6’)-Ie-*
		1	4	R: FOX30, P10,TE30	*tet*M, *tet*K, *mecC, aph(3)-IIIa, msr*A
				I: E15, RD5	
		1		R: FOX 30, P10, TE30	*tet*M, *tet*L, *aph(3)-IIIa*
	*S. lentus*	1	5	R: FOX 30, P10, E15, CN10, TE30	
				I: DA2	
	*S. simulans*	1	3	R: FOX30, P10, E15	
				I: RD5	

1 R – resistant; I – intermediate; ** P10 – penicillin 10 IU; E15– erythromycin 15 mg; DA2 – clindamycin 2 mg; TE30 – tetracycline 30 mg; RD5 – rifampicin 5 mg; QD15 – streptogramin 15 mg; C30 – chloramphenicol 30 mg; FOX30 – cefoxitin 30 mg; CN10 – gentamycin′10 mg; CIP5 – ciprofloxacin 5 mg

**Table 3.** Phenotypic resistance of *Staphylococcus* strains isolated from Polish ewes of the Świniarka and Uhruska breeds

Antimicrobial	*Staphylococcus* spp. n/%		
	*S. aureus* (n = 20)	CoNS (n = 15)	Total
FOX 30*	1/5	11/73.3	12/34.3
C 30*	6/30		6/17.1
CIP 5	1/5		1/6.6
DA 2	6/30	3/20	9/25.7
E15	7/35	4/26.6	11/31.4
CN10	2/10	1/6.6	3/8.5
P10*	6/30	13/86.6	19/54.3
RD 5		2/13.3	2/5.7
QD 15	1/5		1/6.6
TE 30	7/35	7/46.6	14/40

1 CoNS – coagulase-negative species; E15 – erythromycin 15 mg; FOX 30 – cefoxitin 30 mg; CN10 – gentamicin 10 mg; RD5 – rifampicin 5 mg; QD15 – quinupristin/dalfopristin 15 mg; DA2 – clindamycin 2 mg; P10 –penicillin 10 IU; TE30 – tetracycline 30 mg; CIP5 – ciprofloxacin 5 mg; C30 – chloramphenicol 30 mg;

* – statistically significant difference (P-value < 0.05) between resistance to given antibiotic in *S. aureus* and CoNS isolates

### Detection of resistance and virulence genes

The phenotypic resistance of the tested strains was confirmed by the presence of resistance genes; however, no resistance genes were found in as many as 48,4% (n = 15) of strains with phenotypic resistance to at least one tested antimicrobial (n = 31). Resistance to cefoxitin was confirmed by the presence of the *mec*C gene in four cefoxitin-resistant strains (*S. aureu*s, *S. xylosus* and two strains of *S. simulans*) originating from both UHR and SW. In turn, the presence of the *mec*A gene was not detected. Although more than 54% of the strains showed phenotypic resistance to penicillin, the presence of the *bla*Z gene was detected in only two of them (*S. xylosus* and *S. simulans*), and only the *mec*C gene was detected in one additional penicillin-resistant CoNS strain (Table 4).

**Table 4.** Occurrence of resistance genes in *Staphylococcus* strains isolated from Polish ewes of the Świniarka and Uhruska breeds

Resistance gene	*Staphylococcus* spp. %		
	*S. aureus* (n = 20)	CoNS (n = 15)	Total
*acc(6’)-Ie-*	1/5	1/6.6	2/5.7
aph(3)-IIIa*	2/10	6/40	8/22.8
*bla*Z		2/13.3	2/5.7
*cat* (pC194)	3/15	2/13.3	5/14.2
*cat* (pC221)*	5/25		5/14.2
*erm*A	1/5		1/6.6
*erm*B	1/5	1/6.6	2/5.7
*mec*C	1/5	3/20	4/11.4
*msr*A	1/5	1/6.6	2/5.7
*tet*K	1/5	4/26.6	5/14.2
*tet*L	6/30	3/20	9/25.7
*tet*M*		4/26.6	4/11.4

1 CoNS – coagulase-negative species;

* – statistically significant difference (P-value < 0.05) between occurrence of resistance genes in *S. aureus* and CoNS isolates

In the cases of the tetracycline-resistant strains, phenotypic resistance was statistically significantly more common among strains isolated from UHR and was confirmed by the presence of at least one *tet* gene in the majority of the strains (85.7%). Interestingly, the *tetL* gene dominated in the tetracycline-resistant *S. aureus* strains (6 out of 7 isolates) and occurred statistically significantly more frequently among the strains isolated from UHR (Table 5 and Table 6). In the case of the CoNS strains, the *tet*M gene was present with the two other *tet* genes at similar levels, but the presence of this gene was detected only in this group of staphylococci (Table 4). Only two tetracycline-resistant strains did not have any of the analysed genes, while two more strains with phenotypic susceptibility to tetracycline showed the presence of the *tet*M gene, both isolated from SW (Table 6).

**Table 5.** Phenotypic resistance of *Staphylococcus* strains by sheep breed

Antimicrobial	*Staphylococcus* spp. n/%		
	Uhruska (n = 16)	Świniarka (n = 19)	Total
Cefoxitin	4/25	8/42.1	12/34.3
Chloramphenicol*	5/31.3	1/5.3	6/17.1
Ciprofloxacin	1/6.3		1/6.6
Clindamycin	6/37.5	3/15.8	9/25.7
Erythromycin	5/31.3	6/31.6	11/31.4
Gentamicin	2/12.5	1/5.3	3/8.5
Penicillin	8/50	11/57.8	19/54.3
Rifampicin		2/10.5	2/5.7
Streptogramin	1/6.3		1/6.6
Tetracycline*	10/62.5	4/21	14/40

* – statistically significant difference (P-value < 0.05) between resistance to given antibiotic in *Staphylococcus* spp. isolated from Uhruska and Świniarka breed

**Table 6.** Occurrence of resistance genes in *Staphylococcus* spp. by sheep breed

Resistance gene	*Staphylococcus* spp. n/%		
	Uhruska (n = 16)	Świniarka (n = 19)	Total
*acc(6’)-Ie-*	1/6.3	1/5.3	2/5.7
*aph(3)-IIIa*	4/25	4/21	8/22.8
*bla*Z	1/6.3	1/5.3	2/5.7
*cat* (pC194)*	5/31.3		5/14.2
*cat* (pC221)	4/25	1/5.3	5/14.2
*erm*A	1/6.3		1/6.6
*erm*B	2/12.5		2/5.7
*mec*C	2/12.5	2/10.5	4/11.4
*msr*A	1/6.3	1/5.3	2/5.7
*tet*K	4/25	1/5.3	5/14.2
*tet*L*	7/43.7	2/10.5	9/25.7
*tet*M		4/21	4/11.4

* – statistically significant differences (P-value < 0.05) between occurrence of resistance genes in in *Staphylococcus* spp. isolated from Uhruska and Świniarka breed

Almost all strains with phenotypic resistance to chloramphenicol were isolates from UHR (five of the six chloramphenicol-resistant strains) (Table 5) and represented only the *S. aureus* species. In all the cases, phenotypic resistance was confirmed by the presence of the *cat* (p221) and/or *cat* (p194) gene(s). Interestingly, despite the lack of phenotypically identified resistance to chloramphenicol, the presence of the *cat* (p194) gene was detected in two CoNS strains (*S. simulans* strains isolated from UHR). This latter gene predominated statistically significantly among the strains isolated from UHR (Table 6).

Despite the analysis of a relatively wide panel of genes responsible for the phenotype of resistance to macrolides or macrolide-lincosamide-streptogramin B (MLSB), a large percentage of strains with phenotypic resistance to macrolides/lincosamides or streptogramins (n = 15) did not demonstrate the presence of any of the tested genes (n = 11, 73.3%) (Table 2). The *msr*A gene (encoding the macrolide efflux pump) was found only in two erythromycin-resistant strains, while the *erm*A and *erm*B genes (responsible for MLSB) were present in only two erythromycin/clindamycin-resistant isolates, one of which was *S. aureus*. In this strain, both these genes were present at the same time.

Although resistance to kanamycin and neomycin was not tested, the *aph(3)-IIIa* gene was found in as many as eight (22.9%) strains, and most of them (n = 6) were CoNS (*S. xylosus* n = 4 and *S. simulans* n = 2) (P-value < 0.05). In turn, the *acc(6′)-Ie-aph(2″)* gene, encoding the bifunctional enzyme aminoglycoside-6′-N-acetyltransferase/2"-O-phosphoryltransferase responsible for resistance to gentamicin/tobramycin/kanamycin, was demonstrated only in one phenotypically gentamicin-resistant strain of *S. aureus* and in one gentamicin-susceptible strain of *S. xylosus*.

Only a single *S. aureus* strain isolated from UHR showed the presence any gene encoding staphylococcal enterotoxin B (SEB), and this isolate possessed a single such gene. In the case of the remaining strains, none of the virulence determinants tested were detected.

## Discussion

The presence of *Staphylococcus* bacteria in the milk of 70% of the examined lactating sheep may be the result of various processes also related to the occurrence of a pathological condition. The presence of the accepted major pathogens (which include *E. coli, Streptococcus agalactiae* and *S. aureus*) is one determiner of whether a given case is diagnosed as clinical or subclinical mastitis (24). Interestingly, in the current study, the sheep did not present any clinical changes in the udder, which may potentially qualify the *S. aureus*-positive cases as subclinical mastitis, but it should be emphasised that, because sampling followed parturition very closely, the assessment of the number of somatic cells would not have been reliable (3). When CoNS are isolated in the absence of clinical symptoms in the animal, there is a high probability that it may be a subclinical infection, usually with an environmental source, or may merely be carriage of the pathogen. As reported by Vassielieou (29), staphylococcal mammary carriage in ewes is about 6.5%, while the percentage of isolation of CoNS strains in our study was as much as 30%; therefore, it would be difficult to consider all cases as carriers, especially since *S. xylosus* or *S. simulans* (both CoNS), were isolated in over 22% and 14% of the animals, respectively. As shown by Fragkou *et al*. (10), the carrier status in favourable conditions (suppressed animal immunity) may develop into mastitis, especially since clinical mastitis most often develops at the time of weaning as the milking period begins (30). Among the CoNS, different species may be isolated from the udder with different frequencies, depending on the geographical location and the dominant species in the environment (30). *Staphylococcus xylosus* and *S. simulans* were the most frequent species isolated in our study, while they were detected by other researchers at 17 and 9%, respectively, or even in significantly lower counts with only single representatives of these species (2). They also constituted the dominant microbiota of sheep skin (31) and nostrils (18); therefore, their presence may be of environmental origin. Interestingly, *Staphylococcus condimenti* belongs to the staphylococci termed “food associated”; however, like other isolated CoNS species, it may be responsible for invasive infections in humans with reduced immune system efficiency. The number of the CoNS isolates was too low to show a correlation between them and the animal breed, while the number of *S. aureus* isolates was the same in UHR and SW; therefore, it was difficult to indicate the host-species specificity.

The high percentage of *S. aureus* (40%) and CoNS (30%) in the animals may be related to the sheep’s common origin from one farm and the possible direct transmission of strains or species caused by their close contact. One of the causes may be the permanent presence of a specific strain of *S. aureus* on the farm, of which the primary source is not necessarily animals but rather breeders or animal handlers. Generally, the *S. aureus* carriage rate in sheep is relatively low (9–14%), while the carriage rate in breeders is quite high, reaching 60% (23). Permanent contact between animals and the presence of suckling lambs may also be one of the factors predisposing a flock to such a high percentage of *S. aureus* in milk (30). It is related to the potential cross-suckling of other mothers and the spread of the pathogen among animals in this way.

Phenotypic resistance to at least one antimicrobial was recorded in as many as 88.6% of the isolated strains. Among all the isolates, only four *S. aureus* strains (isolated from SW) were susceptible to all the tested antimicrobials, but only one MDR strain was observed in the sheep of this breed. All the isolates from the UHR sheep were resistant, and as many as seven isolates (43% of all the UHR isolates) from both the CoNS group and *S. aureus* were included in the group of MDR strains. However, only in the cases of tetracycline and chloramphenicol was statistically significantly higher resistance observed in the isolates originating from UHR than in those from SW. A similar relationship was also noted for the resistance genes to these antibiotics: *cat* (pC194) and *tet*L, occurred statistically significantly more often in the isolates from UHR than in strains from SW. These results allow us to formulate a cautious hypothesis that the more primitive breed of sheep (SW) may be more resistant to udder colonisation by staphylococci. Because disease incidents are rarer, this breed requires targeted therapy less often, which is associated with the much lower percentage of resistant and multidrug-resistant strains inhabiting the mammary gland of these animals. However, the percentage of positive *Staphylococcus* isolations in both animal breeds was similar (SW 55% *vs* UHR 45%). Additionally, in the case of strains originating from refined breeds, the presence of an MRSA strain was shown, and methicillin resistance was confirmed by the presence of the *mec*C gene. Currently, MRSA strains pose a significant threat to public health and their occurrence in farm animals has increased significantly in recent years, also constituting a source of infection for humans (6). Since MRSA strains may be responsible for causing subclinical mastitis, they are excreted in milk and constitute a potential source of infection for consumers of raw milk or dairy products made from unprocessed milk (6). The occurrence of MRSA in sheep milk was also recorded earlier at a similar, rather low level of only 1.2% (6) or 0.44% (4). Although *mec*C was first demonstrated in 2011 (11), since then it has also appeared as a homologue of the *mec*A gene in MRSA strains from both humans and animals, particularly cattle (4). However, it seems that the occurrence or dominance of the *mec*C or *mec*A gene in a specific herd or even in a given area is related to the spread of a specific type of gene or strain with a specific genotype among animals in close contact with each other (1). Although the occurrence of *mec*C genes in CoNS strains is rare, Loncariac *et al*. (21) demonstrated this type of gene in some *Staphylococcus* species (including *S. xylosus, S. sciuri* and *S. warneri*), the sources of which were both farm animals and wild animals. That the sources were distributed indicates the wide spread of this gene (1). Moreover, it has been proven that genetic elements responsible for methicillin resistance may be transferred between CoNS and CoPS (including *S. aureus*); therefore, CoNS may constitute an important reservoir of beta-lactam resistance (19). However, regardless of the source of CoNS strains, they usually have a high percentage of phenotypic resistance to cefoxitin, as in our study (7).

Generally, although CoNS have been regarded for a long time as less virulent, many researchers consider them a natural reservoir of various resistance mechanisms transferred through horizontal exchange (1). In our study, we confirmed this observation by demonstrating the presence of resistance genes to tetracyclines, aminoglycosides, macrolides and chloramphenicol in many CoNS strains. Interestingly, the gene encoding *aph* (3′)-IIIa (a plasmid-encoded aminoglycoside phosphotransferase) and responsible for resistance to kanamycin and neomycin (16) was also dominant in the CoNS strains. Although this dominance was not statistically significant, these results may confirm the significant role of CoNS strains as reservoirs of resistance genes transferred horizontally. Since the phenotypic resistance to kanamycin and neomycin has not been determined, it is not known whether the presence of the *aph(3)-IIIa* gene is correlated with phenotypic expression of resistance to these antimicrobials, especially in CoNS strains, as four strains belonging to this group also showed the presence of other resistance genes responsible for insensitivity to tetracycline, chloramphenicol and aminoglycosides, including gentamicin (*tet*L, *tet*M, *cat* (pC194) and *acc(6’)-Ie-)* but without their phenotypic expression. This phenomenon was not observed in any of the *S. aureus* strains. Quite interesting is the contrast observed between species: phenotypic resistance to phenicols was detected in over 17% of *S. aureus* isolates and correlated with the presence of one or two resistance genes, while no phenotypic resistance was observed in certain CoNS strains despite their carrying chloramphenicol resistance genes, suggesting lack of gene expression in these strains. The resistance to chloramphenicol is an unexpected phenomenon, because the toxicity of this drug precludes its approval for use in animals and humans; therefore, researchers either did not even analyse this type of resistance (23) or did, but obtained full phenotypic susceptibility of CoNS (31). Both the *cat* (p221) and *cat* (p194) genes encode the production of type A acetyltransferases and are located on the plasmids enumerated in their symbols, which guarantees their rapid spread. They can occur individually on plasmids but often co-occur with other genes encoding resistance to streptomycin or macrolides or are part of a multi-resistance plasmid. These characteristics may partially explain the phenotypic and genetic resistance to chloramphenicol observed in our study despite this drug not having been used in the animals. However, a similar quite high resistance rate of 17% was obtained by other researchers (26).

Resistance to MLS_B_ is the most common resistance mechanism reported in bacteria of the *Staphylococcus* genus; however, there may not be only one mechanism, because MLSb resistance is related to the presence of over 90 different genes (24). We analysed only three genes from the *erm* group, which are most often responsible for this type of resistance. Nevertheless, despite the high percentage of erythromycin (31.4%) and lincomycin-resistant strains (25.7%), only two strains showed the presence of these genes. Resistance in the absence of the three analysed *erm*-group genes can be explained by the presence of numerous other mechanisms encoding this type of resistance. At the same time, only one erythromycin-resistant strain showed the presence of the *msr*A gene encoding macrolide-streptogramin B (MS_B_)-type resistance.

The phenotypic resistance to tetracycline was quite high at 40%, as in studies conducted by other authors (13). Tetracycline is a broad-spectrum antibiotic that has been used for over 80 years; therefore, resistance to tetracycline is common in various species of microorganisms isolated from pets, livestock and wildlife (15). In our study, the dominant gene responsible for tetracycline resistance was *tet*L, present in both *S. aureus* and CoNS (25.7%), while *tet*M appeared only in the CoNS strains. The *tet*K and *tet*L genes encode the efflux pump, while the *tet*M gene encodes another resistance mechanism related to ribosome protection (20). Unlike the other genes mentioned above, *tet*M is also responsible for resistance to minocycline and doxycycline (29). This gene, as indicated by other authors, is mainly associated with the *S. xylosus* species (20), which was also confirmed by our results. In many cases, it is possible to observe the simultaneous occurrence of at least two different genes in the same strain. If they encode two different resistance mechanisms, they may potentially yield a synergistic effect associated with a reduction in the zone of inhibition or an increase in the minimum inhibitory concentration (29).

An interesting phenomenon in our study was the relatively low level of the *bla*Z gene in the genome of penicillin-resistant strains. This gene is most often responsible for encoding penicillinases, associated with phenotypic resistance to beta-lactams. The studies by Aubry *et al*. (5) left confidence in amplification of the *bla*Z gene as the reference method for detecting the presence of penicillinases. Nevertheless, the positive correlation of gene amplification with phenotypic resistance among *Staphylococcus* spp. strains depends largely on the method used to measure drug susceptibility (microdilution method or disc-diffusion method), the *Staphylococcus* species tested and the interpretation criteria used. For example, depending of the bacterial species, the criteria regarding the size of the inhibition zone recommended by EUCAST (the European Committee on Antimicrobial Susceptibility Testing) or the CLSI may correspond better or worse to the presence of the *bla*Z gene. Concurrently, the opposite phenomenon should be taken into account, whereby there may be phenotypic resistance with no parallel genotypic resistance: the *bla*Z gene (like other genes) is not always expressed, because of the relatively high fitness costs for bacteria associated with its expression. Despite the relatively wide pool of analysed virulence genes, especially those of superantigen nature, only one strain of *S. aureus* showed the presence of the SEB enterotoxin encoding gene. Therefore, despite the high potential risk associated with the occurrence of the wide panel of drug resistance, the genome of the tested strains does not indicate that isolated *Staphylococcus* have the status of foodborne pathogens. Similar results were obtained by Gharsa *et al*. (12). It should, however, be borne in mind that in some studies, especially those regarding *S. aureus* isolated from cow’s milk, the range and frequency of occurrence of genes encoding enterotoxins or toxic shock toxin and even Panton–Valentine toxin was significantly higher (17).

## Conclusion

Clinical and subclinical staphylococcal infections currently pose a great diagnostic and therapeutic challenge. Drug-resistant and MDR strains, of which the genomes often contain many genes encoding resistance to several different groups of antimicrobials, easily share this mobilome with other bacterial strains, sometimes even belonging to separate species, through horizontal exchang, causing resistance even without selective pressure. Therefore, unprocessed sheep’s milk may be an important source of highly drug-resistant *Staphylococcus* strains for consumers. The present study highlights the problem of potential subclinical mastitis caused by drug-resistant strains of *S. aureus* and CoNS of the *Staphylococcus* genus among lambed ewes and indicates that the resistance may be related to the selection pressure resulting from frequent application of antibiotic therapy. However, the relationship between the occurrence of drug-resistant *Staphylococcus* strains and the animal breed (domesticated *vs* primitive, *i.e*. more *vs* less resistant to infections and treated more *vs* less frequently), requires further research.
